# Mechanical Power in Endurance Running: A Scoping Review on Sensors for Power Output Estimation during Running

**DOI:** 10.3390/s20226482

**Published:** 2020-11-13

**Authors:** Diego Jaén-Carrillo, Luis E. Roche-Seruendo, Antonio Cartón-Llorente, Rodrigo Ramírez-Campillo, Felipe García-Pinillos

**Affiliations:** 1 Department of Physiotherapy, Universidad San Jorge, Villanueva de Gállego, 30580 Zaragoza, Spain; djaen@usj.es (D.J.-C.); leroche@usj.es (L.E.R.-S.); acarton@usj.es (A.C.-L.); 2 Department of Physical Activity Sciences, Universidad de Los Lagos, 5290000 Osorno, Chile; r.ramirez@ulagos.cl; 3 Department of Physical Education and Sport, University of Granada, 18071 Granada, Spain; 4 Department of Physical Education, Sports and Recreation, Universidad de La Frontera, 4811000 Temuco, Chile

**Keywords:** biomechanics, endurance runners, long-distance athletes, wearable device

## Abstract

Mechanical power may act as a key indicator for physiological and mechanical changes during running. In this scoping review, we examine the current evidences about the use of power output (PW) during endurance running and the different commercially available wearable sensors to assess PW. The Boolean phrases endurance OR submaximal NOT sprint AND running OR runner AND power OR power meter, were searched in PubMed, MEDLINE, and SCOPUS. Nineteen studies were finally selected for analysis. The current evidence about critical power and both power-time and power-duration relationships in running allow to provide coaches and practitioners a new promising setting for PW quantification with the use of wearable sensors. Some studies have assessed the validity and reliability of different available wearables for both kinematics parameters and PW when running but running power meters need further research before a definitive conclusion regarding its validity and reliability.

## 1. Introduction

Endurance running events are on the apex of a performance revolution, with the sub-2-h marathon barrier just broken (i.e., Vienna in 2019). In the same way the power meter changed training and racing in cycling [1] by providing a fair tool to assess performance with accurate replication, it might also change the way runners compete and train.

Power, a term originated in classical physics, is defined as the product of force and velocity [2]. Despite training delivers stress on the body, the way runners measure this level of stress has been very limited. The faster a runner goes, the higher the stress for a certain level of fitness. Training intensity is the true marker to fitness (i.e., capacity to deal with a particular amount of stress) [3]. The application of mechanical load (i.e., external training load factors) and psychological and physiological efforts (i.e., internal training load factors) are affected by training stress [4]. In running, some external load factors including volume and pace are widely used, while physiological internal load factors consider perceived exertion scales, heart rate, or blood lactate level [4]. On multiple training days, running distance alone could overshadow the accumulated training stress and, eventually, misinterpret the overall training stress [4]. Pace might be as clear as volume but, indeed, it is not easy to assess as the running settings (i.e., surface; slope gradient) as well as weather conditions (i.e., wind velocity) or individual internal factors (i.e., stress, sleep, illness) may affect pace considerably and, therefore, challenge pace intensity quantification. None of these variables provides a fair and repeatable method to measure training intensity and, when training stress is measured imprecisely, injury risk may be increased and performance negatively altered. Given that new wearable devices allow to measure external load metrics apart from both volume and pace, there should be a growing focus on a combination of both biomechanical external (i.e., power output (PW)) and internal load metrics in the future of athletes monitoring [4]. 

Running, as cycling, is cyclical in nature. When running, three dimensional movements are needed. Normally, the body describes a forward movement, vertical oscillation, and a bilateral rotation over the running cycle. For such movements, mechanical work is required accounting vertical and forward movements for most of it. Throughout such movements, a runner acquires both kinetic energy and potential energy changes. The applied work runners develop over the loading phase and the subsequent take-off push to lift their body at every stride to work against environmental factors (i.e., ground reaction force, gravity force, and surface) refers to the external mechanical work. Then, the foot absorbs energy when colliding with the ground and produces power when pushing off. During running, expensive equipment such as specific instrumented treadmills [5] have been utilised to acquire force data. Despite their proved accuracy, most coaches and practitioners are forced to avoid their use due to economic issues. 

Over the last years, inertial measurement units (IMUs) emerged, allowing the quantification of performance, providing coaches and athletes an easy-to-use tool to monitor PW during running (e.g., Runscribe (Scribe Lab. Inc., Half Moon Bay, CA, USA), Stryd (Stryd Inc. Boulder, CO, USA) or Myotest (Myotest SA, Sion, Switzerland)). Previous works have demonstrated the direct relationship between anthropometric measures (e.g., body mass) and spatiotemporal parameters and kinetics and kinematics [6,7,8]. Samozino and colleagues [9] attempted to supply an affordable method to assess force-velocity and power-velocity profiles, using anthropometric and spatiotemporal data along over-ground sprint acceleration. However, Samozino’s approach is inapplicable to submaximal velocities. 

Currently, an increasing number of systems allow the assessment of running power (new heart rate monitors by Polar (Polar Electro Ltd., Kempele, Finland) and Garmin (Garmin Ltd., Olathe, KS, USA)). Nevertheless, there is a lack of scientific evidence testing either its validity or reliability, as well as limited insights on the use and interpretation of power in endurance runners, being this reduced to a few books [3,10], and further information provided by the devices’ manufacturers (e.g., Stryd, https://blog.stryd.com/tag/validation-white-papers/; Myotest, https://www.myotest.com/technology; RunScribe, https://runscribe.com/blog/; Stryd, https://blog.stryd.com; Polar: https://www.polar.com/es/smart-coaching/running-power).

Although the validity and reliability of a wide array of wearable sensors have been shown for running spatiotemporal parameters measurement and they seem to be related with PW estimation [11,12,13,14,15], a deeper knowledge on PW in endurance running and a proper understanding on the use of power meters to quantify workload would be an outstanding step forward towards a new boundary within running training and performance. There is a need to measure training intensity with precision and wearable sensors might help monitor the training-induced stress and, although previous review articles have been focused on power data while running [16,17], none of those concentrated on validity and reliability of such wearables for running PW analysis. Advances in the knowledge of endurance running PW would allow the assessment and monitor of power not only in laboratory settings, but in the field as well. Therefore, the aim of this scoping review was to critically examine the available running power meters and the current evidences about their use and application to endurance running performance.

## 2. Materials and Methods

A review of the literature was conducted following the guidelines of the Cochrane Collaboration and taking into consideration the guidance provided by previous studies focused on scoping reviews [18,19]. This design (i.e., scoping review) was selected in order to have a broader approach with the aim of mapping literature characterized by a variety of study designs. Additionally, findings were reported in accordance with the Preferred Reporting Items for Systematic Reviews and Meta-Analyses (PRISMA) for scoping reviews [20].

### 2.1. Eligibility Criteria

Despite the limited evidence on this topic, some a priori inclusion criteria were considered for this scoping review: (i) only peer-reviewed articles were included; (ii) studies that were not published in English were not explored; (iii) no restrictions for age or sex of participants were applied.

Additionally, no limitations regarding the study design were established. All manuscripts related to running with power or power meters were considered, regardless the study design, except literature reviews (e.g., systematic reviews or metanalysis).

### 2.2. Information sources

A systematic search was conducted in the electronic databases PubMed, MEDLINE and SCOPUS for relevant studies until 1 June 2020. Keywords were collected through experts’ opinion, a systematic literature review, and controlled vocabulary (e.g., Medical Subject Headings: MeSH). Boolean search syntax using the operators “AND” and “OR” was applied. The words “endurance”, “running”, “runner”, “power”, and “power meter” were used. Following is an example of a PubMed search: ((((((endurance) OR submaximal) NOT sprint) AND running) OR runner) AND power) OR power meter; Filters: Publication date from 1 January 2000; Humans; English. 

After an initial search, accounts were created in the respective databases. Through these accounts, the lead investigator received automatically generated emails for updates regarding the search terms used. These updates were received on a daily basis (if available), and studies were eligible for inclusion until the initiation of manuscript preparation on 5 June 2020. Following the formal systematic searches, additional hand-searches were conducted. Grey literature sources (e.g., conference proceedings) were also considered if a full-text version was available. In addition, the reference lists of included studies and previous reviews and meta-analyses were examined to detect studies potentially eligible for inclusion. 

### 2.3. Study Selection

In selecting studies for inclusion, the three-step method was followed [21]. The first step, according to this procedure, was an initial restricted search of the appropriate database collection, followed by an analysis of the text words included in the title and abstract, and the index terms used to characterize the document. A second search using all known keywords and index terms was performed through all included databases. Finally, the reference list of all the selected studies and reports has been checked for additional studies. The authors included the aforementioned filters (i.e., the language and the publication date limitations).

### 2.4. Methodological Quality in Individual Studies

To analyse the methodological quality in studies, the recommendations by Cochrane Review Groups were taken into consideration [22]. Since all the studies examined show a cross-sectional design, quality was assessed using the modified version of the Quality Index developed by Downs and Black [23]. The original scale was reported to have good test–retest (*r* = 0.88) and inter-rater (*r* = 0.75) reliability and high internal consistency (Kuder–Richardson Formula 20 (KR-20) = 0.89). The modified version of the Downs and Black Quality Index is scored from 1 to 14, with higher scores indicating higher-quality studies. Two independent reviewers (DJC-FGP) performed this process and, in the event of a disagreement about the methodological quality, a third reviewer (LERS) checked the data and took the final decision on it. Agreement between reviewers was assessed using a Kappa correlation for methodological quality. The agreement rate between reviewers was *k* = 0.93 which can be interpreted as almost perfect [24]. It is worth noting that the study by Snyder and colleagues [25] was excluded as it is a letter to the editor in response to Aubry and colleagues’ [26] work.

## 3. Results

### 3.1. Study Selection

**Figure 1:**
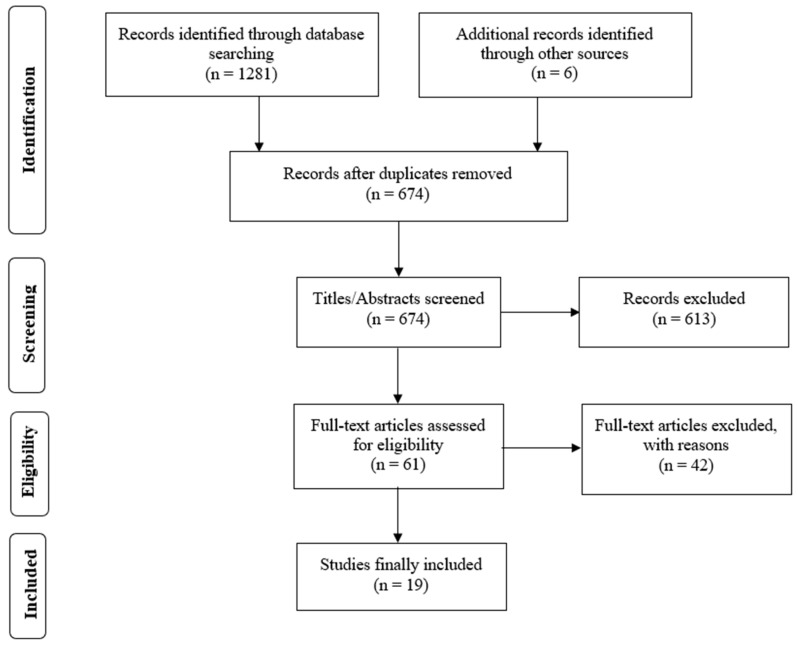
Preferred Reporting Items for Systematic Reviews and Meta-Analyses (PRISMA) flow diagram.

Figure 1 provides a graphical schematization of the study selection process. A total of 1281 studies were initially identified: 640 from PubMed, 378 from SCOPUS, and 263 from MEDLINE. Additionally, 6 studies were identified through other resources. From these 1287 studies, 674 after duplicates removed. The 613 studies excluded after titles and abstracts revisions were essentially based on a lack of relationship with the research interests of this review. After full-text revision, only 19 studies which included either validity or reliability of running wearable sensors suppling running PW and/or the specific discussion of such wearable sensors were considered for the current work. 

### 3.2. Study Characteristics

The main characteristics of the studies included in this review (*n* = 19) are presented in the Table 1 and Table 2. Table 1 shows a summary of 12 studies using wearable sensors with the capacity of measuring power during different running exercises. Whereas three of those studies [11,27,28] examine the PW kinetics during different running protocols, the other four studies [15,25,26,29] investigate the relationship between PW and physiological parameters such as oxygen consumption (VO_2_) at different intensities. Additionally, two further works [30,31] analyse the application of mathematical models, based on power laws, to predict running performance, whereas a recent study [32] assesses the agreement level between two mathematical models and five power meter devices through different running conditions. Other studies examined some parameters provided by the RunScribe power meter to describe the effects of the fatigue induced over a marathon [33,34] and the influence of different types of ankle treatments on running biomechanics [35].

The closest agreement of the Stryd and PolarV technologies with the TPW1 and TPW2 models suggest these tools as the most sensitive, among those analysed, for PW measurement when changing environments and running conditions

Table 2 summarises the studies (*n* = 7) focused on the validity and reliability analysis of kinetic and kinematic parameters for different wearable sensors with the capacity to measure power. Of note, no studies have examined the concurrent validity of PW during running estimated from any power meter, finding only two studies [12,15] which examined the reliability of PW during running. The remaining 5 studies tested the validity and reliability of spatiotemporal parameters [11,14], kinematic parameters [37,38], or both variables [13].

Table 3 shows the methodological quality of the studies examined. Once the review studies and the letter to editors were excluded, 18 studies were assessed with this purpose. Out of a total score of 14 points, all studies reported from 11 to 14 points. Of note, 16 out of 17 studies reported 0 in the item 12 (i.e., participants prepared to participate representative of entire population) and 14 out of 17 studies reported 0 in the item 23 (i.e., randomised).

## 4. Discussion

This review provides a critical assessment on the existing scientific literature regarding PW quantification in endurance running as well as the different current accessible devices for its estimation. After the meticulous analysis described above, a few studies aiming at assessing running power in relation to physiological parameters and power-duration relationship at several running intensities were found. Eighteen studies included in this review were assessed in order to determine the methodological quality and high scores were reported according to the modified Downs and Black scale [23] (i.e., all studies reported more than 11 points out of a total score of 14). Although no studies attempting to assess concurrent validity of PW estimation in running using power meters, their reliability for such estimation was analysed. 

The controversy surrounding power estimation in running is rooted in the question of whether it is indeed power which is being estimated. Unlike cycling, running entails negligible external mechanical work. It involves positive and negative work; the former, pushing off with each stride and the latter, braking on landing [39]. Moreover, elastic energy stored in the Achilles tendon and other tissues makes a significant contribution as up to fifty percent of power required for each step is released as these tissues stretch upon landing and subsequently recoil to aid pushing off. The issue when estimating power in running is that even perfect estimates do not closely correlate to effort required [39]. During cycling, the relationship between mechanical power and total metabolic energy consumption remains constant when conditions are altered, but this is not so when running [39,40]. Readers need to be aware that given the recent application of power meters to endurance running, the increasing need for PW quantification, and the consequent novelty of this research interest, the limited information available might make the discussion of the current study difficult. However, the subsequent sections seek to provide some insight into how running power quantification can help enhance running performance and its quality. 

### 4.1. Current Evidence on PW during Running

While in cycling PW is measured in reference to both direction and quantity of the force applied to the crank, as well as its angular velocity, power needs to be calculated in a different way while running. Since forward and vertical movements of the body account for most of the mechanical work, an accurate calculation of both horizontal and vertical power over the propulsion phase (i.e., a function of forward force and vertical force, respectively) is required to measure running power effectively.

Mechanical power on flat terrain might be estimated in mechanical terms just as function of runner anthropometry (height, mass), spatiotemporal parameters (speed, step rate, ground contact time) and wind speed employing model proposed recently by Jenny and Jenny [41]. In steady running on flat surface, mechanical power and the rate of mechanical energy dissipated into heat should match. 

Considering this assumption and following the mathematical approach mentioned above [41], mechanical energy in steady flat running compiles the energy dissipated by aerodynamic drag, dissipation due to both vertical oscillation and braking. The aerodynamic contribution may be estimated based on air and runner density and running and wind velocity. However, when running on a treadmill wind speed can be considered zero reducing, thus, the importance of this variable. 

On the one hand, dissipation in vertical oscillation can be estimated regarding step rate, ground contact time, running velocity and a potential energy recovery factor. This factor is variable between subjects and that might be the main concern with this assumption. The lack of considering this factor could lead to overestimation in this part of the mechanical power. On the other hand, dissipation due to braking ground reaction force could be modelled by using the runner’s centre of mass excursion and spring-mass model assumptions. In that context, the power generated in a horizontal direction to maintain running velocity could be estimated by anthropometrics, running speed, and the aforementioned energy recovery factor. 

The most controversial part of such a model [41] might be the energy recovery factor. Nevertheless, when measuring mechanical power calculations employing ‘gold standard’ methods different assumptions are, done making the assessment of mechanical PW a challenging measure even in the best testing conditions. 

The critical power (CP) in tasks such as swimming, cycling, and running and its relationship with VO_2_, blood lactate threshold, and work-exhaustion time was critically reviewed by Vandewalle and colleagues [42]. Theoretically, CP supposes the existence of a particular work-rate that can be held before exhaustion [43]. In this review [42], it is determined that CP matches a steady state during heavy submaximal exercises (i.e., between 6 and 30 min). On the contrary, CP is not a reliable predictor of exhaustion time considering the hyperbolic nature of power-exhaustion time relation [42]. Another review focused on the existing models for residual performance capacity estimation and its application for pacing [16]. The authors examined the quantity of work than can be executed in exercises above CP. Although the review by Vandewalle and colleagues found CP to be a poor predictor of exhaustion time given the power-exhaustion time relation, Jones and Vanhatalo determined that within a range of various exercise intensities (e.g., endurance running), this relationship gives a fundamental basis to proper understand the physiological bases of fatigue development, what may result in an outstanding effect for monitoring both training and athletic performance [16].

The power-duration relationship was also described over a wide range of power intensities [17]. Three different exercise intensities were identified. First, exercise intensity below aerobic threshold (i.e., fatigue appears slowly and it mainly has a central origin) was defined as moderate intensity. Then, intensity over lactate threshold but under CP was referred as heavy intensity (i.e., there is a depletion of muscle glycogen due to central and peripheral fatigue). Finally, severe intensity was identified referring to an intensity above the CP, which relates to gradual muscle metabolic homeostasis alterations and subsequent peripheral fatigue [17]. Literature shows different calculation methods for power-duration relationship such as power law [44,45] and hyperbolic models [46,47,48], and exponential decay operations [49,50]. Seemingly, hyperbolic calculations of power-duration relation suit best for both reasonable physiological estimations and a proper option to the fundamental data [17] but, the truth is that all these calculations are operationally weak for coaches and extremely time-consuming. In order to counteract the models mentioned above and to provide in-field application for running biomechanics monitoring and training loads tracking to clinicians, coaches and practitioners, wearable technologies were upgraded considerably and made economically affordable. A review study on wearable devices and their provided metrics (i.e., kinetic and kinematic parameters) in the evaluation and treatment of runners identified best practices, applications and potential limitations of such systems [51]. The author stated that clinicians should assure that the use of wearable sensors should be based on evidence aiming at running-related injuries prevention and performance enhancement, and the guidelines given by each sensor’s manufacturer must be followed [51].

Regarding evidence-based use of wearable sensors, the relationship between VO_2_ as metabolic demand and running PW measured by five commercially available technologies was recently assessed [15]. Twelve endurance-trained male athletes completed 10 submaximal multistage running tests wearing a portable metabolic computer. On two occasions (test-retest), the athletes performed three submaximal treadmill running protocols with manipulations in speed, body weight and slope, and the same protocol was repeated in an athletic track. The Stryd system showed the higher concurrent validity to the VO_2_ (r ≥ 0.911) between the five wearables, and it was also found as the more repeatable and sensitive in all the conditions studied. Furthermore, the level of agreement between these 5 wearable systems was also analysed against two physics theoretical models for PW estimation [10,52] in different running conditions [32], showing that the Stryd and Polar Vantage systems are the most sensitive tools for PW estimation in running given their close agreement with both theoretical models (r > 0.93). The Stryd power meter estimates power production while running separating this metric into two parts: power and form power. Apparently, power reflects the PW associated with changes in the athlete’s horizontal movement, while form power represents the power production originated by the combination of the oscillatory up and down movements of the centre of mass and lateral power as the athlete moves forward. This system utilises mathematical calculations to estimate these two parameters from kinematic data collected from the described movements executed by the runner’s foot [29]. Form power apparently represents the power production originated by the combination of the oscillatory up and down movements of the centre of mass and lateral power as the athlete moves forward. 

On the other hand, the power-VO_2_ relationship in elite and recreational runners had been previously assessed by Aubry and colleagues [26]. To this aim, 13 amateur and 11 elite runners executed a two-setting protocol (i.e., indoor and outdoor). Indoors, participants developed 3 sequential paces (i.e., elite: 14, 16, and 18 km·h^−1^; amateur: 11−16 km·h^−1^) 2 min each, where VO_2_ was analysed via gases expiration system. Outdoors (no precipitations and minimal wind), participants were asked to run at the same pace that they ran indoors. Participants ran for 4 min each pace while measuring VO_2_ using a portable metabolic computer. Additionally, Stryd was used to calculate running power in both settings. Regarding the relationship metabolic demand-running power, the authors found a significant but weak correlation between VO_2_ and running power (*r* = 0.29, *p* = 0.02). Comparing both settings, metabolic demands were found to be significantly higher (i.e., greater VO_2_) outdoors (i.e., outdoor track) than when treadmill running. When speed increased, the difference in VO_2_ values become higher amongst treadmill and outdoor running [26]. Then, after assessing metabolic demand-running mechanics relationship, the authors found moderate strength associations for metabolic demand and ground contact time, vertical oscillation, and step frequency at treadmill running in recreational runners [26]. The authors of the aforementioned study concluded that the use of Stryd power meter should be avoided when assessing running economy as it is unable to distinguish the metabolic demands of an athlete when running on different settings (i.e., outdoors vs. indoor). Of note, the version used during the study is not mentioned (the latest version is even able to consider air resistance) limiting, therefore, their findings. Controversially, Snyder and colleagues clarified several important methodological mistakes made by Aubry and colleagues [26] which led to confusing conclusions [25]. Regarding surface, VO_2_ was measured long before steady state for treadmill tests (latest VO_2_ test started at 1:30 min), but much later over ground (latest VO_2_ test started at 3:30). It is well-known, as stated by Snyder and colleagues, that VO_2_ needs more than 1:30 min to reach steady state causing, therefore, great differences between VO_2_ when measured at 1:30 and 3:30 min, and, even greater at faster speeds [25]. The authors claimed that these methodological flaws exclude precise correlation analysis between VO_2_ and power measured with Stryd on different surfaces [25]. Considering speed, a speed-normalised power to speed-normalised VO_2_ correlation was reported in the article [26], therefore denying VO_2_ change because of speed [53]. Snyder and colleagues [25] suggested the use of the accepted physiological term ‘cost of transport’ instead of ‘metabolic demand’, which was used by the authors and leads to confusion in the readers and it does not vary over speed [54]. The actual power-VO_2_ correlation is proposed to address this error [25]. With respect to subjects, Snyder and colleagues [25] criticise the individual assessment of training metric as they [26] collect data by subject prior executing the correlation analysis when within-subject correlation between VO_2_ and further variables is appropriate for training and racing [55]. For such study [26], data collection should be developed over different within-subject measurements [25].

Furthermore, the Stryd reliability for PW during treadmill running at a self-selected constant speed with a slope gradient at 0% was proved to be a stable data between short and long intervals (i.e., 10–120 s and 180 s, respectively) [28]. No significant differences were found in the amount of power production between the different spans of times acquired (*p* = 0.276, partial ETA^2^ = 0.155) and an almost perfect association in the previously mentioned amount of power production recorded over the intervals (ICC ≥ 0.999). As the authors mentioned, the conditions in which the study was performed may influence the stability of running power over time and these findings should not be taken for granted when transferred to over-ground running [28]. The findings reported here seem to be very advantageous for clinicians and practitioners since, if compared to other physiological parameters such as heart rate or VO_2_, PW tend to stabilise over time earlier than others traditionally used. However, PW is a mechanical parameter which considers work per time. That work exhibits a muscular and tendinous component. While muscle work needs oxygen consumption to produce work, tendons store and release energy without consuming any oxygen. Therefore, work produced while running requires different quantities of oxygen depending upon the amount of work is done by muscles or tendons. Thus, PW may not be directly related to running metabolic cost. Following the evidence-based use of wearable sensors, it has been found a linear power-velocity relationship(*r* = 0.999) at submaximal speed, and, the consequent used of the two-point method to predict PW in running at different speeds using the Stryd power meter [36]. The authors executed an incremental run-to-exhaustion protocol on a motorized treadmill at 0% slope gradient. The power-velocity relationship determined from three two-point methods at proximal (10 and 12 km·h^−1^), intermediate (10 and 14 km·h^−1^), and distal (10 and 17 km·h^−1^) speeds showed the same precision than the multiple-point method (used also by the authors to compare PWs through the study) to provide PW estimated by the Stryd power meter. As stated by the authors of the aforementioned study, since the two-point method can be developed faster and without developing fatigue in the athletes, it should be used when assessing PW to acquire accurate power estimations over a range of submaximal running speeds [36]. This might be an outstanding contribution to the strength and conditioning scene as the power-velocity relationship could be frequently updated influencing, therefore, on the quality of both running training and performance. The lack of evidence regarding the power-biomechanics (i.e., contact time, flight time, step frequency, step length, surface) relationship as well as the effect of fatigue on PW when running expose the need of further research on how the running gait parameters and environmental factors affect PW estimation. Bridging the gap between research and practical use of power in running would bring the stunning potential of such parameter to light. The insights provided here into the validity and reliability of the different commercially available wearable sensors for spatiotemporal parameters show the emerging potential of such devices for running PW measurement given their narrow association considering theoretical approaches previously proposed [6,7,8,9].

### 4.2. Commercially Available Systems to Measure PW during Running

Despite the application of IMUs for estimating PW during running being recent, different commercially systems are available. Two of the most widely used wearable sensors for such purposes are Stryd and Runscribe. 

Stryd system is a pioneer in manufacturing wearable power meters for running. Stryd estimates running power in watts. This power meter, a foot pod reinforced with carbon fibre (weight: 9.1 g) and based on an IMU of 6 different axis (i.e., 3-axis accelerometer and 3-axis gyroscope) and with a sampling rate of 1000 Hz, attaches to the runner’s shoe to estimate metrics for performance quantification (i.e., pace and distance, average elapsed power, maximal power, average elapsed form power, average elapsed leg spring, and average elapsed ground time). Some studies have analysed the reliability of this sensor for both spatiotemporal and PW parameters [11,12,15]. Of note, the latest version of Stryd is capable of estimating the energy expenditure of working against air resistance by measuring the air resistance one faces while running in regards with a white paper located at the manufacturer’s website and where the trials performed to assess the Stryd’s ability to determine wind speed are meticulously described (https://storage.googleapis.com/stryd_static_assets/white_papers/wind-white-paper-8-17.pdf). This sensor employs both kinematic and environmental microelectromechanical sensors together with user-supplied biometrics and proprietary physical and data-driven algorithms to calculate air resistance force as follows:

(1) $$F_{A}\;=\;\frac{1}{2}\rho C_{d}Av^{2}$$

where *ρ* stands for air density, *C_d_* for drag coefficient, *A* for the cross-sectional area that encounters the air resistance, and *v* for the vector of the runner’s relative velocity with local air mass surrounding them. According to the aforementioned white paper, the Stryd system should be centrally located on the laces and towards the toe of the shoe as this placement reported the lowest error regarding wind measurement accuracy (i.e., wind technology is able to correctly report relative air speed under 4 km·h^−1^). However, no peer-reviewed research has been performed to assess the level of accuracy of such device when accounting for air resistance arising therefore the need to evaluate it in the near future. 

The use of the Runscribe wearable sensor attached to either the lace or heel of the shoes, based on a nine-axis (three-axis magnetometer, accelerometer, and gyroscope, respectively) IMU with an accuracy of 0.002 seconds (sampling rate: 500 Hz), is also widespread around the running world. The way Runscribe estimates power is based on GOVSS model [52] and various assumptions. GOVSS model estimates power using the runner’s speed, step rate, weight, and height, as well as slope gradient and wind velocity based on linear regression models [52]. Several studies attempted to determine the reliability and validity of such foot pods for either kinetic or kinematic parameters [13,14,15,37,38].

Despite the common use of the Stryd and Runscribe wearable sensors, there are other options for running power estimation commercially available. Cerezuela-Espejo and colleagues [15] also analysed Garmin Running Power (v1.6, Olathe, KS, USA) and Polar Vantage V (firmware 3.1.7, Polar, OY, Kempele, Finland). The Garmin device estimates PW data derived from the combination of a Garmin sport watch and one of the sensors recommended by the manufacturer (i.e., HRM-Run or HRM-Tri heart rate monitor and Running Dynamics Pod on the waist belt). Polar Vantage V estimates power production with no need of an extra sensor (e.g., foot pods). This multisport watch is capable of calculate indirectly several metrics such as average power, maximum power and laps power using the built-in barometer and GPS sensors. Although a positive relation with VO_2_ was found for both devices (*r* ≤ 0.841), they exhibited limited test-retest reliability, particularly Garmin Running Power in laboratory settings and Polar Vantage V outdoors. Myotest device, usually fixed onto a belt and fastened and placed level with the navel’s runner (according to manufacturer’s guidelines), provides, amongst others (i.e., cadence, runner’s centre of mass vertical movement, contact time, flight time, step length, stiffness, pace, distance), running PW. Unfortunately, the way Polar, Garmin, and Myotest estimate PW remains unrevealed.

Every wearable sensor that provides power metrics employs some form of running power model combined with different assumptions. Therefore, there exist conditions in which such models do not concur until all the different wearable sensors standardise and implement the same model for running PW estimation.

### 4.3. How Valid and Reliable is PW during Running When Measured by These Devices?

Despite the lack of a concurrent validity study where any of the commercially available power meters are compared with the ‘Gold Standard’ to measure running power (i.e., force-plate-instrumented treadmill or a long force platform system), the accuracy of the PW when running provided by these wearable devices might be limited. The variety of available technologies for running gait analysis (e.g., accelerometers, gyroscopes, force plates, pressure plates, and photoelectric cells) implies a variety of devices should exist for analysing stride characteristics. However, some of these devices have not been validated yet. The validity and reliability of a gait analysis system are essential to determine whether results are due to changes in gait pattern or are simply systematic measurement errors. As already mentioned, white (non-peer-reviewed) papers provided by manufacturers to promote the likely potential of their devices, attribute the different values of running power obtained by the different devices to differences in estimating power. Indeed, Myotest attempted to demonstrate validity and repeatability of Myotest App on an Apple watch for PW analysis in comparison with Garmin-Garmin Pod, Polar Vantage V, Stryd (White paper provided by the manufacturer, https://www.myotest.com/technology). A sample of 7 runners executed a 2000 m run protocol with an elevation gain of 22.8 m where 500 m were run on flat ground, 500 m uphill at a constant slope, 500 m of constant-slope downhill, and 500 m on flat ground at a self-selected speed over the entire protocol. It was reported that given the outputs shape and the existence of similar peaks, a correlation between the analysed systems is seemingly demonstrated considering that the different systems are sensitive to elevation changes (i.e., lower power at uphill/downhill shift and higher power at uphill running). Mean-normalised power signal was used to remove the constant shift in the signals, and it was shown that PW measured with Myotest is closer to power measured with Garmin and Stryd. These findings must be taken cautiously as it is well-known that white papers lack the peer-review process.

Concerning the reliability of such wearables, a recent study analysed the repeatability different devices (Stryd, Runscribe, Garmin Running Power, and Polar Vantage V) show when measuring power when running as well as their concurrent validity against VO_2_ [15]. For such a purpose, 12 highly-trained endurance runners executed a submaximal incremental running speed test and a submaximal incremental body weight test in two different settings (i.e., outdoor and indoor). An additional increasing slope gradient at submaximal speed test was executed only indoor. After completion, the authors found Stryd to be the most repeatable device for power estimation. Additionally, Stryd concurrent validity assessment for power estimation was found to show the closest relationship with the VO_2max_ measured directly by metabolic cart [15]. Of note, the authors of this study distinguish between Stryd App and Stryd Watch. Although the Stryd sensor is found to be the same using both app and watch, the variations reported by the authors between these systems is not justified. It might be arguable that the normalisation applied by each system (i.e., Stryd app and watch) differs from one other, but this is not mentioned by the authors. Nevertheless, the findings reported by Cerezuela-Espejo and colleagues [15] constitute a huge contribution providing clinicians, coaches, and practitioners a reliable wearable sensor to quantify running power in training, retraining, and competition.

Some of these devices have been used previously for measuring running kinetics (i.e., PW amongst others) and kinematics parameters (i.e., running spatiotemporal gait characteristics). The aforementioned GOVSS model [52] and Jenny’s model [41] for estimating mechanical power rely mainly on runners anthropometry, environmental factors (i.e., air density and wind speed) and running spatiotemporal parameters (i.e., speed, step rate and ground contact time). With this in mind the measurement of spatiotemporal parameters is essential for an accurate power estimation. Regarding this, some studies have shown good reliability of wearable sensors when measuring such parameters [11,12,13,14,15]. García-Pinillos and colleagues [11], over a speed incremental running protocol on a treadmill, tested the reliability of Stryd for running spatiotemporal parameters (i.e., contact time, flight time, step length, and step frequency) against a proved reliable photoelectric cell system for such purpose (i.e., Optogait system) [56]. The authors found that Stryd measures accurately step length and step frequency but underrates slightly contact time overrates flight time in comparison with such system. Likewise, the intra-Stryd reliability has also been analysed [12] over two different 5-min tasks (i.e., two self-paced walks along a trail a and two trail runs separated by a 5-min rest period) with 20 healthy individuals (it was not mentioned whether the participants had any running experience). The authors assessed all the data provided by the Stryd power meter. Regarding trail running, all variables were found to have relative test-retest reliability, meeting the set the intraclass correlation coefficient (ICC) threshold. When considering an interval of confidence equals to 95%, pace, average elapsed power, average elapsed form power, average elapsed leg spring, and vertical oscillation were deemed to have good to excellent reliability; maximal power, average elapsed ground time, and distance were reported to exhibit moderate to excellent reliability [12]. 

The intra-validity analysis of the Runscribe sensor has also been examined [13,14]. This sensor was used to measure spatiotemporal (i.e., contact time, step length, and cycle time), kinematic (i.e., foot pronation excursion and pronation velocity), and kinetic parameters (i.e., impact ground force and braking ground force) on two different surfaces (i.e., track and grass) at two different running speeds (comfortable self-selected speed and an increased speed) [13]. Over two 1600-m runs, first at a slow pace and then fast on two randomised-ordered surfaces (i.e., track and grass), Runscribe foot pod sensors were found to be valid to determine variations in the aforementioned spatiotemporal, kinetic, and kinematic parameters in different conditions (i.e., different surfaces) [13]. Furthermore, validity measurements regarding the Runscribe placement on the running shoes have also been examined [14]. In this study, the location of the Runscribe on the running shoes (i.e., heel or shoelace) was assessed against a reference technology (i.e., high-speed video camera at 1000 Hz). The authors found Runscribe to be a valid system to examine spatiotemporal variables in treadmill running. Additionally, the location of the Runscribe needs to be considered as it was found to be sensitive to metrics accuracy. When analysing contact time, flight time, and step length, the shoelace placement is recommended as smaller errors were found when comparing to the Runscribe attached to the heel. In contrast, the heel showed higher accuracy when analysing step frequency [14]. In a recent study [15] where test-retest reliability of several wearable sensors was tested, Runscribe was found to be the second most repeatable sensor for speed, slope gradient, and body weight (standard error of measurement ≥ 30.1 W, coefficient variation [CV] ≥ 7.4%, ICC ≤ 0.709), only after the Stryd power meter, for indoor settings. However, when employed in outdoor, Runscribe exhibits both the highest errors and poorest repeatability (SEM ≥ 59.3 W, CV ≥ 14.8%, ICC ≤ 0.563) [15]. When its concurrent validity between PW estimation and VO_2_ consumption examined over an increasing speed test by a metabolic cart, Runscribe exhibited values of *r* ≥ 0.582 and standard error of estimate (SEE) ≤ 13.7% for indoor and outdoor settings. Moreover, the power estimation and VO_2_ agreement was reduced over both conditions (body weight, SEE = 10.3%; slope, SEE = 18.5%). Regarding data collection, it is worth highlighting that the authors did not specify the placement of the Runscribe wearable sensors affecting, as previously discussed, the possible interpretation of the measured outcomes.

## 5. Conclusions

The previous works on running PW and the theoretical approaches provided for its estimation are, from a practical standpoint, hard to include in the everyday routine of an athlete. This study provides a critical evaluation of available scientific information regarding PW quantification in endurance running as well as the different accessible devices for its estimation. The inexistence of studies attempting to evaluate concurrent validity of PW estimation measured by wearable sensors when running (apart from non-peer-reviewed manufacturer’s white papers), the limited available information about the dynamic of PW during running and its short-term response to acute influencing factors (e.g., velocity, slope, fatigue) and long-term training adaptations (i.e., PW as a tool for monitoring training adaptations) made the analysis reported here especially difficult. However, it is arguable that the outcomes stated here are tremendously useful as PW stabilises earlier than other variables commonly used (i.e., heart rate or VO_2_). Furthermore, running power increases alongside velocity, resembling their linear relationship at different submaximal speeds. Additionally, the reliability of commercially available wearables has been assessed, finding Stryd to be the most reliable and accurate wearable device for running PW estimation. Ultimately, given their novelty and potential application, the analysis of PW while running and its estimation by wearable devices needs more attention from a research perspective in order to provide practitioners a reliable, valid, and friendly tool to improve both training and performance quality in running.

## Figures and Tables

**Table 1:** Studies (*n* = 12) involving the use of wearable sensors with the capacity of measuring power during running protocols.

References	Subject Description	Aim	System Used	Protocol	Outcome Measures	Results
Dobrijevic et al. (2017) [15]	30 physical education students (15 men and 15 women)	To explore the properties of the F-V relationship of leg muscles exerting the maximum pulling F at a wide range of V on a standard motorized treadmill	Motorized treadmill using externally fixed strain gauge dynamometer (CZL301, ALL4GYM, Serbia) connected to the subject wearing a wide and hard weightlifting belt	Walking and running on a treadmill at different velocities (1.4−3.3 m.s^−1^), and maximum pulling F exerted horizontally were recorded	Leg muscle capacities for producing maximum F, V, and power	The F-V relationship of leg muscles tested through a wide range of treadmill V could be strong, linear, and reliable. Moreover, the two-velocity method could provide reliable and ecologically valid indices of F, V, and P producing capacities of leg muscles.
García-Pinillos et al. (2019) [17]	49 endurance runners	To examine how the PW changes while running at a continuous comfortable velocity on a motorized treadmill by comparing running power averaged during different time intervals	Stryd system (foot pod)	Runners performed a 3 min running protocol at comfortable velocity and P was examined over six recording intervals within the 3-min recording period: 0−10 s, 0−20 s, 0−30 s, 0−60 s, 0−120 s and 0−180 s	Running PW	P during running is a stable metric with negligible differences, in practical terms, between shorter (i.e., 10, 20, 30, 60 or 120 s) and longer recording intervals (i.e., 180 s)
Aubry et al. (2018) [14]	24 male runners (13 recreational, 11 elite)	To investigate the applicability of running power (and its individually calculated run mechanics) to be a useful surrogate of metabolic demand (Vo_2_), across different running surfaces, within different caliber runners.	- Stryd system (chest strap) - Gas exchange measures (Cosmed Quark CPET and Cosmed K5 systems)	2 different test at 3 different paces, while wearing a Stryd on both an indoor and an outdoor test: -Treadmill vO_2_ test: running at 3 speeds for 2 min each -Outdoor vO_2_ test (on track): identical speeds for 4 min (1 min rest)	- Spatiotemporal parameters - Running PW - vO^2^	Running power (with Stryd) is not a great reflection of the metabolic demand of running in a mixed ability population of runners
Snyder et al. (2017) [13]	Manuscript clarification: Request for clarification to Aubry et al. (2018)					Some major methodological flaws in the mentioned paper are detected. The authors concluded that data analysis and, thereby, data interpretation are misleading
Austin et al. (2018) [18]	17 well-trained distance runners	To measure the correlations between running economy and P and form power at LT pace.	- Stryd system (foot pod) - Gas exchange measures (Parvo Medics TrueOne 2400)	Participants ran two 4 min trials: one with a self-selected cadence, and one with a target cadence lowered by 10%	- Gas exchange measures - RPE - Power - Form power - SF	RE is positively correlated with Stryd’s power and form power measures yet the footpod may not be sufficiently accurate to estimate differences in the running economy of runners
García-Pinillos et al. (2019) [36]	18 recreationally-trained male endurance runners	To determine if the P-V relationship in endurance runners fits a linear model when running at submaximal velocities, as well as to examine the feasibility of the “two-point method” for estimating P at different velocities	Stryd system (foot pod)	Incremental running protocol on a treadmill. Initial speed was set at 8 km.h^−1^, and speed increased by 1 km.h^−1^ every 3 min until exhaustion	PW (W)	The two-point method based on distant velocities was able to provide P with the same accuracy than the multiple-point method.
Vandewalle et al. (2018) [21]	Data from 6 elite endurance runners	- To apply the P-law and logarithmic models and four asymptotic models to the individual performances of the elite runners. - To compare the accuracy of these models. - To compare the predictions of MAS by interpolation and the prediction of maximal running speeds for long distances by extrapolation	-	The empirical models were compared from the performance of 6 elite endurance runners who participated in international competitions over a large range of distances	Mathematical models to predict running performance	The predictions of long-distance performances (maximal running speeds for 30, 60 min and marathon) by extrapolation of the logarithmic and power-law models were more accurate than the predictions by extrapolation in all the asymptotic models.
Mulligan et al. (2018) [20]	Data from various records for a range of distances	To develop a novel, minimal and universal model for human running performance that employs a relative metabolic P scale	-	European and world records performances for eight distances, from 1 km to the marathon, were analyzed	Mathematical models to predict running performance	The model presented provides a quantitative method for extracting characteristic parameters from race performances of runners. This is the to date most accurate theoretical description of running performances that does not require any a priori fixing of physiological constants
Gregory et al. (2019) [25]	12 young adults with history of ankle sprain	RunScribe system (foot pod, on the heel)	To evaluate the effects of ankle taping, bracing, and fibular reposition taping (FRT) on running biomechanics	Four 400 m runs at self-selected pace on an outdoor track. Each run was performed in a different condition (control, taped, braced, FRT)	- Spatiotemporal (CT, CycleT, SL) - Kinematic (PR, PR_veloc_) - Kinetic (impact G, braking G)	Ankle taping and bracing were shown to be comparable in decreasing ankle kinematics and kinetics, while FRT caused minimal changes in running biomechanics
Leuchanka et al. (2019a) [23]	15 endurance runners	To examine the changes in spatiotemporal variables during a marathon race	RunScribe system (foot pod, on the lace shoe)	Monitoring spatiotemporal variables over a marathon race by comparing 3 points (km 5, 26 and 37)	- Spatiotemporal (Pace, CT, SL and cadence)	Significant differences were found in pace, SL, and CT when compared across 3 race points
Leuchanka et al. (2019b) [24]	15 endurance runners	To measure the kinematic asymmetry during a marathon race	RunScribe system (foot pod, on the lace shoe)	Monitoring kinematic variables over a marathon race by comparing 3 points (km 5, 26 and 37)	- Kinematic variables for right and left foot (pace, strike index, PR, PR_veloc_)	Changes in asymmetry were not found to be statistically significant over the marathon.
Cerezuela-Espejo et al. (2020) [22]	10 endurance runners	To analyse agreement level between power estimated PW by five commercial wearable systems and two theoretical models in different environments and conditions	5 systems: - Stryd App - Stryd Watch - RunScribe (foot pod) - Garmin Running P (watch and chest strap) - Polar Vantage (watch)	Three submaximal running protocols on a treadmill (indoor) and an athletic track (outdoor), with changes in speed, body weight, and slope.	Running PW derived from the 5 systems and theoretical PW from two mathematical models (TPw1 and TPw2).	The closest agreement of the Stryd and PolarV technologies with the TPW1 and TPW2 models suggest these tools as the most sensitive, among those analysed, for PW measurement when changing environments and running conditions

CP: critical power; LT: blood lactate thresholds; Vo2_max_: maximal oxygen uptake; t_lim_: exhausting time at a given intensity; W´: residual performance capacity; F: force; V: velocity; P: power; D_lim_: exhaustion distance; MTT: Montreal Track Test; MAS: maximal aerobic speed; CT: ground contact time; SL: step length; PR: pronation excursion; PRveloc: pronation velocity; TPw1: Mathematical model for power output (PW) estimation 1; TPw2: Mathematical model for PW estimation 2; PW: power output.

**Table 2:** Studies (*n* = 7) examining the reliability and validity of different wearable sensors with the capacity to measure power during running.

References	Subject Description	Tested System	Reference System	Protocol	Outcome Measures	Results
García-Pinillos et al. (2018) [16]	18 trained endurance runners	Stryd system (foot pod)	OptoGait system	Incremental running test (8−20 km·h^−1^ with 3-min stages) on a treadmill	- Spatiotemporal parameters (CT, FT, SL, SF)	Stryd is reliable for measuring spatiotemporal parameters. It provides accurate SL and SF measures but underestimates CT (0.5−8%) and overestimates FT (3−67%)
Koldenhoven et al. (2018) [32]	12 recreational runners	RunScribe wearable sensor	3D motion capture system (Vicon system)	2.4 km running protocol on treadmill, at self-selected speed	- PR, PR_veloc_, and CycleT	RunScribe showed good to excellent concurrent validity for the outcome measures
Brayne et al. (2018) [31]	13 runners	Wireless accelerometer (RunScribe): skin mounted	Uniaxial piezoresistive accelerometer (model 352C22, PCB Piezotronics): skin mounted	Participants ran at 3 different speeds on a treadmill (2.5, 3.5, 4.5 m.s^−1^) for a total of 40 s (10 s to regulate running gait and 30 s data collection)	- Peak tibial acceleration (g)	RunScribe accelerometer accurately measures peak tibial accelerations when compared to a research accelerometer, at a range of speeds
Hollis et al. (2019) [33]	15 recreational runners	RunScribe system (foot pod, on the heel)	Intra-system comparison (in different experimental conditions)	Two 1600 m runs (slow: 3−4; fast: 5−6 on a 0−10 RPE scale) on two surfaces (track, grass). Randomized order.	- Spatiotemporal (CT, CycleT, SL) - Kinematic (PR, PR_veloc_) - Kinetic (impact G, braking G)	RunScribe sensor is valid to identify changes in the outcome measures when participants ran in different conditions.
Navalta et al. (2019) [29]	20 young, healthy individuals	Stryd system (foot pod)	Intra-system reliability	Two 5 min self-paced walks along a trail, and two 5 min trail runs (5 min rest period)	- Pace and distance - Power: average elapsed power, maximal power, average elapsed form power - Stiffness: average elapsed leg spring - Spatiotemporal: CT - Vertical oscillation	Trail running task returns moderate to excellent reliability across all measures
García-Pinillos et al. (2019) [30]	49 amateur endurance runners	RunScribe system (foot pod) on 2 locations: - Heel shoe - Lace shoe	High-speed video analysis at 1000 Hz	Treadmill running for 3 min at self-selected comfortable velocity	- Spatiotemporal gait parameters (CT, FT, SL, SF)	RunScribe is a valid system to measure spatiotemporal parameters during running on a treadmill. The location of the RunScribe plays an important role on the accuracy of spatiotemporal parameters. The lace shoe placement showed smaller errors for CT, FT and SL, whereas the heel shoe was more accurate for SF
Cerezuela-Espejo et al. (2020) [19]	12 endurance-trained male athletes	5 systems: - Stryd App - Stryd Watch - RunScribe (foot pod) - Garmin Running P (watch and chest strap) - Polar Vantage (watch)	- Metabolic cart (VO_2_)	Participants were initially familiarized with the protocol and then, two protocols were performed in two different settings (outdoor vs. indoor): - Testing 1: Submaximal protocol with incremental speed - Testing 2: Submaximal protocol with incremental body weight A 3rd testing condition was performed only indoor, with increasing slope at submaximal velocity	- P output during running	The Stryd system is the most repeatable technology, among the five analyzed, for P estimation. The concurrent validity analysis indicated that PW estimated by the Stryd device showed the closest relationship with the VO_2_ directly measured by the metabolic cart.

CT: ground contact time; CycleT: cycle time; SL: step length; PR: pronation excursion; PR_veloc_: pronation velocity; RPE: rate of perceived exertion; FT: flight time; SF: step frequency; VO_2_: oxygen uptake; RE: running economy; PW: power output.

**Table 3:** Modified Downs and Black scale [23].

Study	Item 1	Item 2	Item 3	Item 6	Item 7	Item 10	Item 12	Item 15	Item 16	Item 18	Item 20	Item 22	Item 23	Item 25	Total (out of 14)
Dobrijevic et al. (2017) [15]	1	1	1	1	1	1	1	1	1	1	1	1	0	1	13
Aubry et al. (2018) [14]	1	1	1	1	1	1	0	1	1	1	1	1	0	1	12
Austin et al. (2018) [18]	1	1	1	1	1	1	0	1	1	1	1	1	0	1	12
García-Pinillos et al. (2019) [36]	1	1	1	1	1	1	0	1	1	1	1	1	0	1	12
García-Pinillos et al. (2019) [17]	1	1	1	1	1	1	0	1	1	1	1	1	0	1	12
Vandewalle et al. (2018) [21]	1	1	1	1	1	1	0	U	1	1	1	0	0	1	11
Mulligan et al. (2018) [20]	1	1	1	1	1	1	0	U	1	1	1	0	0	1	11
Gregory et al. (2019) [25]	1	1	1	1	1	1	0	1	1	1	1	1	1	1	13
Leuchanka et al. (2019a) [23]	1	1	1	1	1	1	0	1	1	1	1	1	0	0	11
Leuchanka et al. (2019b) [24]	1	1	1	1	1	1	0	1	1	1	1	1	0	0	11
García-Pinillos et al. (2018) [16]	1	1	1	1	1	1	0	1	1	1	1	1	0	1	12
Koldenhoven et al. (2018) [32]	1	1	1	1	1	1	0	1	1	1	1	1	0	1	12
Brayne et al. (2018) [31]	1	1	1	1	1	1	0	1	1	1	1	1	1	1	13
Hollis et al. (2019) [33]	1	1	1	1	1	1	0	1	1	1	1	1	0	1	12
Navalta et al. (2019) [29]	1	1	1	1	1	1	0	1	1	1	1	1	0	1	12
García-Pinillos et al. (2019) [30]	1	1	1	1	1	1	0	1	1	1	1	1	0	1	12
Cerezuela-Espejo et al. (2020) [19]	1	1	1	1	1	1	0	1	1	1	1	1	1	1	13
Cerezuela-Espejo et al. (2020) [22]	1	1	1	1	1	1	0	1	1	1	1	1	1	1	13

Key: 0 = no; 1 = yes; U = unable to determine. Item 1: clear aim/hypothesis; Item 2: outcome measures clearly described; Item 3: patient characteristics clearly described; Item 6: main findings clearly described; Item 7: measures of random variability provided; Item 10: actual probability values reported; Item 12: participants prepared to participate representative of entire population; Item 15: Blinding of outcome measures; Item 16: analysis completed was planned; Item 18: appropriate statistics; Item 20: valid and reliable outcome measures; Item 22: participants recruited over same period; Item 23: Randomised; Item 25: adjustment made for confounding variables.
